# Strawberry Intake Ameliorates Oxidative Stress and Decreases GABA Levels Induced by High-Fat Diet in Frontal Cortex of Rats

**DOI:** 10.3390/antiox8030070

**Published:** 2019-03-20

**Authors:** Cuauhtémoc Sandoval-Salazar, Cecilia I. Oviedo-Solís, Edmundo Lozoya-Gloria, Herlinda Aguilar-Zavala, Martha S. Solís-Ortiz, Victoriano Pérez-Vázquez, Cristina D. Balcón-Pacheco, Joel Ramírez-Emiliano

**Affiliations:** 1 Departamento de Enfermería y Obstetricia, División de Ciencias de Salud e Ingenierías, Campus Celaya-Salvatierra, Universidad de Guanajuato, Celaya 38060, Mexico; cuauhtemocss@gmail.com (C.S.-S.); linda_az99@hotmail.com (H.A.-Z.); 2 Departamento de Medicina y Nutrición, Universidad de Guanajuato, León 37672, Mexico; cioviedo@outlook.com; 3 Laboratorio de Bioquímica y Biología Molecular de Productos Naturales de Plantas, CINVESTAV, Irapuato 36821, Mexico; edmundo.lozoya@cinvestav.mx; 4 Departamento de Ciencias Médicas, División de Ciencias de la Salud, Campus León, Universidad de Guanajuato, León 37320, México; silviasolis17@prodigy.net.mx (M.S.S.-O.); vicpe@yahoo.com (V.P.-V.); dorianybalcon@gmail.com (C.D.B.-P.)

**Keywords:** high-fat diet, rat frontal cortex, γ-aminobutyric acid, oxidative stress

## Abstract

It has been proposed that there is a correlation between high-fat diet (HFD), oxidative stress and decreased γ-aminobutyric acid (GABA) levels, but this has not been thoroughly demonstrated. In the present study, we determined the effects of strawberry extract intake on the oxidative stress and GABA levels in the frontal cortex (FC) of obese rats. We observed that an HFD increased lipid and protein oxidation, and decreased GABA levels. Moreover, UV-irradiated strawberry extract (UViSE) decreased lipid peroxidation but not protein oxidation, whereas non-irradiated strawberry extract (NSE) reduced protein oxidation but not lipid peroxidation. Interestingly, NSE increased GABA concentration, whereas UViSE was not as effective. In conclusion, our results suggest that an HFD increases oxidative damage in the FC, whereas strawberry extract intake may ameliorate the disturbances associated with HFD-induced oxidative damage.

## 1. Introduction

A sedentary lifestyle and unhealthy food habits are considered the main factors leading to obesity [1]. For example, hypercaloric diets containing large amounts of refined sugars and saturated fats produce brain damage and systemic oxidative stress [2], which is likely to disrupt important processes such as food intake, but the mechanisms are not well understood. It is important to note that adequate concentrations of a neurotransmitter such as γ-aminobutyric acid (GABA) are needed for effective neurotransmission, which is needed to regulate several conditions and processes like food intake [3]. Animal models have been used to elucidate how hypercaloric diets affect brain function and processes such as food intake [4]. GABA is the main inhibiting neurotransmitter in mammals’ brains, and as such is implicated in the control of neural excitability, plasticity and synchronization, as well as the modulation of the release of neurotransmitters [5]. Likewise, GABA is implicated in the regulation of food intake in the hypothalamus [3]. In this regard, rats fed a high-fat diet (HFD) for 9 weeks showed decreased GABA concentrations in the dorsomedial hypothalamus [6]. Another study showed that mice harboring a deletion of vesicular GABA transporter in leptin receptor neurons were prone to develop obesity with higher food intake and lower energy expenditure [7]. Therefore, it is probable that decreased GABA release affects the inhibitory control of food intake in the frontal cortex (FC).

In obese humans, the disinhibition and asymmetry of the FC seems to favor the development of eating disorders and anxiety, which are linked to binge eating [8]. Also, damage in the FC was shown to increase the risk of excessive food intake [9]. Current research lines relate the FC with the control of inappropriate situations that must be inhibited, for example, during the selection of a food type that will be eaten, leading to an HFD. An HFD may lead to the development of obesity, which is associated with increased oxidative stress in both humans and animal models [10]. Oxidative stress produces cellular dysregulation, increased production of proinflammatory molecules, energy imbalance and an increased risk of type 2 diabetes, hypertension, hyperlipidemia and brain damage [11]. For these reasons, it is necessary to propose strategies to enhance antioxidant defenses and decrease oxidative stress, preventing the complications of obesity.

Strawberries contain phytochemicals with potent antioxidant and anti-inflammatory properties, such as anthocyanins, caffeic acid, ellagic acid and flavonoids including tannins, catechin, quercetin, kaempferol and gallic acid derivatives. They also contain vitamins C, E and carotenoids [12,13]. It has been demonstrated that dietary supplementation with the antioxidant curcumin reduces oxidative stress [14,15] and reduces brain damage by increasing levels of the brain-derived neurotrophic factor (BDNF) in obese and diabetic mice [16]. Interestingly, berry diets increase the expression of the neuroprotective trophic factor insulin growth factor-1 (IGF-1) in rat brain, suggesting that berries are potent regulators of the brain signaling aimed at enhancing cognitive function [17]. Strawberry intake was also shown to reduce obesity and improve glycemic control in experimental animals fed an HFD [18]. A study showed that neurochemical changes occurred in the FC and hippocampus of rats exposed to ^56^Fe, which increased inflammation and oxidative stress; a strawberry diet showed significant amelioration of neurotoxicity induced by ^56^Fe irradiation [19]. Similarly, it was reported that strawberry extracts are scavengers of free radicals [20], and the ellagic acid isolated from strawberries prevented the oxidation of low-density lipoprotein (LDL) induced by the proliferation of rat aortic smooth muscle cells [21]. Moreover, dietary supplementation with 50 g freeze-dried strawberry powder (2 cups of strawberry drink per day) to women with metabolic syndrome was shown to decrease lipid peroxidation and cholesterol levels [22]. Together, these data suggest that the ellagic acid and flavonoids in strawberries play a beneficial role in human health.

Irradiation with ultraviolet light (UV) increases antioxidant and polyphenol contents in fresh-cut fruits [23]; however, high doses of UV can produce oxidation of the bioactive compounds [24]. Therefore, the effectiveness of UV radiation depends on factors such as dose, light source, species and cultivar [25]. In this respect, Ayala-Gil et al. described that UV irradiation increased the concentration of phenolic compounds and various antioxidants in strawberries [25]. Thus, the aim of this study was to evaluate the effect of HFD on GABA levels and oxidative stress, as well as to determine the antioxidant capacity of UV-irradiated and non-irradiated strawberry extracts in HFD-fed rats.

## 2. Materials and Methods 

### 2.1. Production of Strawberry Extract

The strawberries (*Fragaria* × *ananassa*) to produce the strawberry extract (SE) were purchased from local producers at Irapuato, Gto, México. Thus the SE was produced as we previously described [25,26]. First, the strawberries were sanitized and sliced (6 mm thick). Second, these slices were irradiated with ultraviolet light-C (UV-C) at 1.2 W/m^2^/16.5 min, and other ones were used without irradiation. Third, the slices were frozen at −20 °C followed by lyophilization over 7 days. Fourth, lyophilized slices were ground, then 1 g of this ground was mixed with 20 mL of methanol:acetic acid (100:1 *v/v*) for 2 h at 5 °C, followed by centrifugation at 500 rpm for 30 min. Fifth, the supernatant was concentrated in a rotary evaporator (BÜCHI 461) at 39 °C, whereas the pellet was mixed with 20 mL of acetone:acetic acid (100:1 *v/v*) for 2 h at 5 °C and then concentrated in a rotary evaporator at 39 °C. Sixth, the samples concentrated from methanol and acetone were mixed and dissolved together in distilled water. Seventh, the UV-irradiated and non-UV-irradiated aqueous extracts were incubated at a ratio of 2:1 with 2N HCl in a water bath at 100 °C for 1 h, following by cooling and centrifugation at 1200 *g* for 30 min. After, the samples were incubated at a ratio of 1:1 with ethyl acetate for 2 min, then the organic phase was recovered. Finally, the UV-irradiated and non-UV-irradiated organic extracts were concentrated in a rotary evaporator at 39 °C, followed by mixing with starch for better handling.

### 2.2. Animal Care and Strawberry Extract Treatment

Male Wistar rats (21 month-old, and weight: 100–150 g) were randomized into four groups. The control group consisted of 5 rats fed a standard diet (named as SD; Purina Rodent Chow, Purina Mexico: 28.5% protein, 13.5% lipids and 58% carbohydrates); the second group included 5 rats fed a high-fat diet (named as HFD; Purina Chow, Purina Mexico: 17% protein, 47.5% lipids and 35.5% carbohydrates); the third group consisted of 5 rats fed an HFD supplemented with non-irradiated strawberry extract 0.2% (named as HFD-NSE) and finally, the fourth group were 5 rats fed an HFD supplemented with UV-irradiated strawberry extract 0.2% (named as HFD-UViSE). The SD and HFD groups were fed for 20 weeks without receiving any treatment. The HFD-NSE and HFD-UViSE groups were first fed an HFD for 12 weeks, then these groups were fed an HFD supplemented with NSE and UViSE, respectively, for eight weeks. The four groups had ad libitum access to water and food.

At the end of the 20-week treatment, the rats were sacrificed by cervical dislocation and quickly the brain FC was dissected and homogenized in a cold buffer (10 mM HEPES, 0.6% Nonidet p-40, 150 mM NaCl, 1 mM EDTA) containing protease inhibitors (Complete, Boehringer Mannheim, Germany) and 0.1% of the antioxidant curcumin (70% purity; Sigma-Aldrich; Saint Louis, MO 63103, USA. cat. no. 1386) before being stored at −30 °C. Whole homogenized FCs were assayed for protein concentration using bicinchoninic acid (BCA) protein assay (Pierce Chemical Co., Rockford, IL, USA) with bovine serum albumin (BSA) as the reference standard. All animal procedures were conducted in accordance with the 1996 Guide for the care and use of laboratory animals (Institute of Laboratory Animal Resources 1996) [27] and Mexican legislation (NOM-062-ZOO-1999) [28]. The study was approved by the Institutional Ethical Committee from the University of Guanajuato (Reg. no. CIBIUG-P-38-2015).

### 2.3. Measurement of Lipid Peroxidation and Oxidized Proteins

Oxidation of polyunsaturated fatty acids generates reactive aldehydes like malondialdehyde (MDA) and 4-hydroxyalkenals, which were quantified with the thiobarbituric acid-reactive substances (TBARS) assay using 150 µg of whole homogenized FC; whereas oxidation of proteins generates carbonyls that were measured using 300 µg of whole homogenized FC as previously was described [14].

### 2.4. GABA Level Determination

One hundred micrograms of whole homogenized FC were used to determine GABA levels, which were quantified using HPLC-UV detection and an acetonitrile:water (35:65, *v/v*) mobile phase as we previously described [4].

### 2.5. Statistical Analysis

Statistical analyses were performed with Statistics for Windows 8 (StatSoft, Inc., Tulsa, OK USA). The ANOVA and Kruskal–Wallis results analogous to non-normal data followed by Tukey’s post hoc test were used to find differences between groups. The significance level was set at *p* < 0.05. Spearman’s correlation coefficient (ρ) was used to analyze associations between GABA levels and oxidative damage markers using IBM-SPSS version 20 (IBM, Armonk, NY, USA). 

## 3. Results

### 3.1. Effect of Strawberry Extracts on Oxidative Damage in the Brain

**Figure 1:**
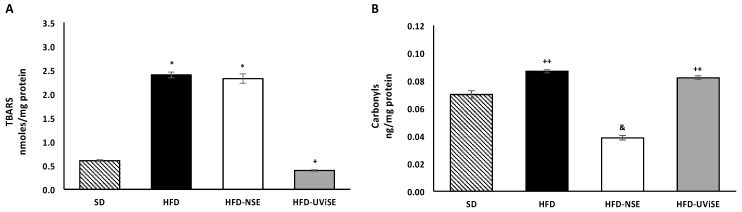
Effects of strawberry extracts on oxidative damage in the frontal cortex. (**A**) Lipid peroxidation represented by thiobarbituric acid-reactive substances (TBARS) levels, and (**B**) protein oxidation represented by carbonyls levels. SD, standard diet; HFD, high-fat diet; HFD-NSE, high-fat diet + non-irradiated strawberry extract; HFD-UViSE, high-fat diet + irradiated strawberry extract. Data are given as the mean ± standard error of the mean (SEM). * *p* = 0.0001 vs. the SD and HFD-UViSE groups; + *p* = 0.03 vs. the SD group; ++ *p* = 0.009 vs. the SD group; & *p* = 0.0001 vs. the SD, HFD and HFD-UViSE groups.

Figure 1A shows that in the FC, TBARS levels were higher in the HFD and HFD-NSE groups compared with the SD group (*p* = 0.0001), whereas these levels were lower (*p* = 0.03) in the HFD-UViSE group compared with the HFD gorup, even below the SD group. 

With respect to oxidized protein levels in the FC of the HFD and HFD-UViSE groups, the protein carbonyl levels were significantly different from those of the SD group (*p* = 0.009). Interestingly, in the HFD-NSE group, the protein carbonyl levels were lower (*p* = 0.0001) than in the SD, HFD and HFD-UViSE groups (Figure 1B).

### 3.2. A High-Fat Diet Decreases GABA Levels in the Frontal Cortex and Hippocampus

**Figure 2:**
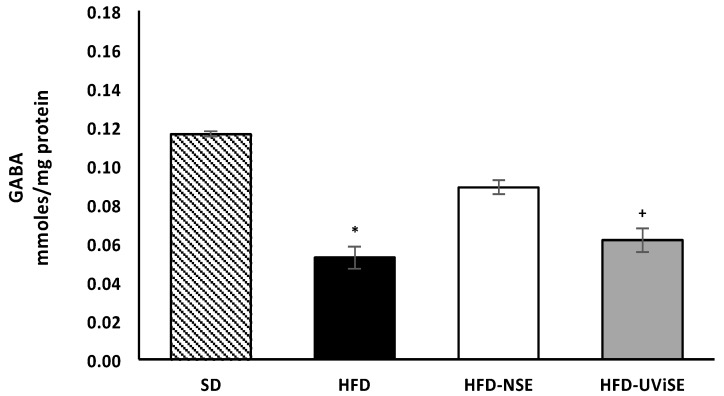
Effects of strawberry extracts on GABA levels in the frontal cortex. SD, standard diet; HFD, high-fat diet; HFD-NSE, high-fat diet + non-irradiated strawberry extract; HFD-UViSE, high-fat diet + irradiated strawberry extract. Data are given as the mean ± standard error of the mean (SEM). * *p* < 0.005 vs. the SD and HFD-NSE groups; + *p ≤* 0.001 vs. the SD and HFD-NSE groups.

Figure 2 shows significantly decreased GABA levels in the FC of HFD-fed rats with respect to the SD and HFD-NSE groups (*p* = 0.0001 and *p* = 0.001, respectively). The GABA levels in the HFD-UViSE group were lower compared to those in the SD and HFD-NSE groups (*p* ≤ 0.001) and showed no difference compared with the HFD group.

### 3.3. Correlation between Oxidative Damage and GABA Levels

**Figure 3:**
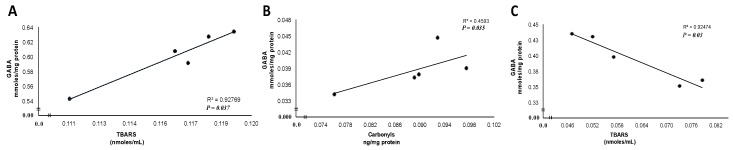
The relationship between GABA and TBARS levels in the SD group (**A**) and HFD-UViSE group (**C**), and the relationship between GABA and carbonyl levels in the HFD-NSE group (**B**).

Finally, the data were analyzed in order to determine whether GABA levels correlate with the markers of oxidative damage. GABA levels were positively correlated with TBARS levels in the FC of the SD group (Figure 3A), whereas GABA levels positively correlated with carbonyl levels in the FC of the HFD-NSE group (Figure 3B). Interestingly, in the HFD-UViSE group, GABA levels were inversely correlated with TBARS levels (Figure 3C).

## 4. Discussion

Strawberries are a source of dietary polyphenols and vitamins [12,13,29]. For these reasons, strawberries play an important beneficial role by improving antioxidant defenses against the development of several chronic diseases [30,31]. The present results show that, as predicted, HFD increased lipid and protein oxidation and decreased GABA concentration in the rat FC. This is consistent with previous reports where decreased GABA levels and increased oxidative damage were described in the FC of HFD-fed obese rats [2,4].

To reduce oxidative damage in the FC of obese rats, extracts were obtained from UV-irradiated and non-irradiated strawberries, and these were orally administrated to obese rats. It is important to mention that UV-irradiation increases the production of phenolic compounds, flavonoids, anthocyanins, fisetin and pelargonidine in the SE. Moreover, the antioxidant capacity was higher in UViSE than in NSE as we previously described [25,26]. The present results show that in the HFD-UViSE group the TBARS levels were similar to those in the SD group, suggesting that UV-irradiation increased the antioxidant content of the strawberry, but this extract did not reduce carbonyl levels; meanwhile, NSE reduced carbonyl levels but not TBARS levels. These results are consistent with results that we described previously; first, the effect of UV-irradiation on the antioxidant content of the strawberry was previously described and second, the effect of extracts was similar to those observed in the cerebellum [26]. A study performed in women with metabolic syndrome reported a significant reduction of malondialdehyde (MDA) levels after treatment with a strawberry drink for four weeks [22]. Similarly, SE reduced the membrane lipid peroxidation in dermal fibroblast [32] and inhibited increased-oxidative stress in 3T3L1 cells by suppressing intracellular reactive oxygen species (ROS) production and decreasing TBARS content. Likewise, superoxide dismutase (SOD) and catalase (CAT) activities and gene expressions were increased [33]. Moreover, diet supplementation with fruits rich in antioxidant polyphenols reduced lipid peroxidation; thus, in rats with hypercholesterolemia the strawberry polyphenols reduced MDA levels to be similar to those shown in normo-cholesterolemic rats [34]. Beneficial effects of strawberry methanolic extract were observed in HepG2 cells, where this extract decreased total cholesterol, LDL and triglyceride levels while stimulating the p-AMPK/AMPK, LDL receptor, sirtuin 1 (Sirt1) and peroxisome proliferator activated receptor gamma coactivator 1-alpha (PGC-1α) expression compared to the control [35]. Another study that used ethanol-induced gastric lesions in rats showed that strawberry polyphenols attenuated the MDA concentration in injured gastric mucosa, indicating that SE can attenuate the process of lipid peroxidation [36].

With respect to the protein oxidation, we found that the HFD increased the FC protein oxidation compared to the SD group, whereas NSE treatment decreased carbonyl content in the FC of rats fed an HFD. These data are in accordance with previous reports that suggest that strawberry has potential beneficial effects on obesity, metabolic syndrome and neurological diseases [29]. Moreover, a study demonstrated that HFD increased the protein carbonyls in the hippocampus, and induced declines in cognitive performance [37]. Together these data suggest that strawberry supplementation could reduce oxidative stress in the brain and have a protective role, which can be related to the antioxidant and anti-inflammatory effects of the strawberry.

It has been proposed that the increased oxidation of proteins and lipids likely alters the membrane cell and damages the organelles and protein structures, reducing neurotransmitter release [38]. Also, it was described that there is a correlation between HFD intake, neurotransmitter concentration and damage in the rat brain. Studies in rats suggested that HFD significantly reduces glutamate uptake and induces a reduced synaptic efficacy in the rat hippocampus [39,40,41]. In the present study, the analysis of correlation showed that GABA levels were positively correlated with TBARS levels in the FC of the SD group, and with the carbonyl content in the HFD-NSE group where the NSE did not have any effect. This suggests that HFD induces oxidative damage, and cells increase GABA levels to correct the alteration in the GABA signal pathway. In addition, GABA levels were inversely correlated with TBARS levels in the FC of the HFD-UViSE group, suggesting that UViSE decreased lipid peroxidation. Consequently, GABA levels were decreased because the harmful stimulus was partially corrected. Therefore, the present results suggest that NSE could prevent the oxidation of important structures that play a key role in GABA metabolism and/or in the signal pathway of this neurotransmitter, such as membrane lipid peroxidation.

We found decreased GABA concentration in the FC of rats fed an HFD, which could be detrimental to oxidative glucose metabolism and the synthesis of neurotransmitters (i.e., glutamate and GABA). This could cause homeostatic dysregulation between neurons and glial cells like microglia [41]. Pathological changes in ventromedial prefrontal cortex (vmPFC) function impair the control of food intake and may facilitate eating disorders, obesity and other disorders of appetitive motivation [42]. Moreover, other authors suggested that the consumption of high-caloric diets generates oxidative stress and memory loss [43], decreases serotonin transporters in the human hypothalamic region [44] and oxidation to lipids and proteins in the brain [43] and probably impairs the release of neurotransmitters. In the present study, NSE treatment increased GABA concentration compared to the HFD and HFD-UViSE groups, likely because the extract reduced the inflammation and oxidative damage, resulting in the protection of the GABA signaling pathway. Moreover, it has been described that flavonoids can increase membrane fluidity, because some can bind to the membrane surface predominantly via hydrogen bonds while others can insert into the lipid bilayer [45].

Therefore, the present data and those reported previously suggest that SE may reduce oxidative damage and prevent the reduction of GABA levels, improving the cognition and control of food intake. This is supported by a model of aged rats fed with a diet supplemented with 2% blueberry, in which a retardation of cognitive decline and neural function was shown to occur by a reduction of nuclear factor kappa B (NF-kB) expression in the FC [46]. Moreover, it was described that the blueberry diet was able to improve cognitive performance in rats with inflammation induced by the central administration of kainic acid, likely by reducing the expression levels of IL-1beta, TNF-alpha and NF-kB as well as increasing the expression of the neuroprotective trophic factor IGF-1 [17]. Finally, the present results show that UViSE decreased lipid peroxidation but not protein oxidation, whereas NSE had a contrary effect. Likely this is due to the change in concentration of the different polyphenols contained in the extracts, or the generation of different polyphenols induced by the UV-treatment. However, the present study did not characterize all the different polyphenols.

## 5. Conclusions

In conclusion, in the present study, HFD increased the lipid and protein oxidation and decreased GABA levels. Moreover, UViSE decreased lipid peroxidation but not protein oxidation whereas NSE reduced protein oxidation but not the lipid peroxidation. Interestingly, NSE increased GABA concentration, but UViSE was not as effective. Our findings suggest that the intake of natural products such as strawberries may be a strategy to prevent the brain damage associated with obesity and to increase lowered GABA levels.

## References

[1] Han J.C., Lawlor D.A., Kimm S.Y. (2010). Childhood obesity. Lancet.

[2] Freeman L.R., Haley-Zitlin V., Rosenberger D.S., Granholm A.C. (2014). Damaging effects of a high-fat diet to the brain and cognition: A review of proposed mechanisms. Nutr. Neurosci..

[3] Delgado T.C. (2013). Glutamate and GABA in appetite regulation. Front. Endocrinol..

[4] Sandoval-Salazar C., Ramírez-Emiliano J., Trejo-Bahena T., Oviedo-Solís C.I., Solís-Ortiz M.S. (2016). A high-fat diet decreases GABA concentration in the frontal cortex and hippocampus of rats. Biol. Res..

[5] Olsen R.W., DeLorey T.M. (1999). GABA and glycine. Basic Neurochemistry: Molecular, Cellular and Medical Aspects.

[6] Abreu A.R., De Abreu A.R., Santos L.T., de Souza A.A., da Silva L.G., Chianca D.A., de Menezes R.C. (2014). Blunted GABA-mediated inhibition within the dorsomedial hypothalamus potentiates the cardiovascular response to emotional stress in rats fed a high-fat diet. Neuroscience.

[7] Vong L., Ye C., Yang Z., Choi B., Chua S., Lowell B.B. (2011). Leptin action on GABAergic neurons prevents obesity and reduces inhibitory tone to POMC Neurons. Neuron.

[8] Ochner C.N., Green D., van Steenburgh J.J., Kounios J., Lowe M.R. (2009). Asymemetric prefrontal cortex activation in relation to markers of overeating in obese humans. Appetite.

[9] Lenard N.R., Berthoud H.R. (2008). Central and peripheral regulation of food intake and physical activity: Pathways and genes. Obesity.

[10] Furukawa S., Fujita T., Shimabukuro M., Iwaki M., Yamada Y., Nakajima Y., Nakayama O., Makishima M., Matsuda M., Shimomura I. (2004). Increased oxidative stress in obesity and its impact on metabolic syndrome. J. Clin. Investig..

[11] Ye Z.W., Zhang J., Townsend D.M., Tew K.D. (2015). Oxidative stress, redox regulation and diseases of cellular differentiation. Biochim. Biophys. Acta.

[12] Giampieri F., Alvarez-Suarez J.M., Tulipani S., Gonzàles-Paramàs A.M., Santos-Buelga C., Bompadre S., Quiles J.L., Mezzetti B., Battino M. (2012). Photoprotective potential of strawberry (Fragaria × ananassa) extract against UV-A irradiation damage on human fibroblasts. J. Agric. Food Chem..

[13] Kårlund A., Salminen J.P., Koskinen P., Ahern J.R., Karonen M., Tiilikkala K., Karjalainen R.O. (2014). Polyphenols in strawberry (Fragaria × ananassa) leaves induced by plant activators. J. Agric. Food Chem..

[14] Martínez-Morúa A., Soto-Urquieta M.G., Franco-Robles E., Zúñiga-Trujillo I., Campos-Cervantes A., Pérez-Vázquez V., Ramírez-Emiliano J. (2013). Curcumin decreases oxidative stress in mitochondria isolated from liver and kidneys of high-fat diet-induced obese mice. J. Asian Nat. Prod. Res..

[15] Jiménez-Flores L.M., López-Briones S., Macías-Cervantes M.H., Ramírez-Emiliano J., Pérez-Vázquez V. (2004). A PPARγ, NF-κB and AMPK-dependent mechanism may be involved in the beneficial effects of curcumin in the diabetic db/db mice liver. Molecules.

[16] Franco-Robles E., Campos-Cervantes A., Murillo-Ortiz B.O., Segovia J., López-Briones S., Vergara P., Pérez-Vázquez V., Solís-Ortiz M.S., Ramírez-Emiliano J. (2014). Effects of curcumin on brain-derived neurotrophic factor levels and oxidative damage in obesity and diabetes. Appl. Physiol. Nutr. Metab..

[17] Shukitt-Hale B., Lau F.C., Carey A.N., Galli R.L., Spangler E.L., Ingram D.K., Joseph J.A. (2008). Blueberry polyphenols attenuate kainic acid- induced decrements in cognition and alter inflammatory gene expression in rat hippocampus. Nutr. Neurosci..

[18] Prior R.L., Wu X., Gu L., Hager T.J., Hager A., Howard L.R. (2008). Whole berries versus berry anthocyanins: Interactions with dietary fat levels in the C57BL/6J mouse model of obesity. J. Agric. Food Chem..

[19] Poulose S.M., Bielinski D.F., Carrihill-Knoll K.L., Rabin B.M., Shukitt-Hale B. (2014). Protective effects of blueberry- and strawberry diets on neuronal stress following exposure to 56Fe particles. Brain Res..

[20] Wang S.Y., Jiao H. (2000). Scavenging capacity of berry crops on superoxide radicals, hydrogen peroxide, hydroxyl radicals, and singlet oxygen. J. Agric. Food Chem..

[21] Chang W.C., Yu Y.M., Chiang S.Y., Tseng C.Y. (2008). Ellagic acid suppresses oxidised low-density lipoprotein-induced aortic smooth muscle cell proliferation: Studies on the activation of extracellular signal-regulated kinase 1/2 and proliferating cell nuclear antigen expression. Br. J. Nutr..

[22] Basu A., Wilkinson M., Penugonda K., Simmons B., Betts N.M., Lyons T.J. (2009). Freeze-dried strawberry powder improves lipid profile and lipid peroxidation in women with metabolic syndrome: Baseline and post intervention effects. Nutr. J..

[23] Alothman R., Bhat R., Karim A.A. (2009). UV radiation-induced changes of antioxidant capacity of fresh-cut tropical fruits. Innov. Food Sci. Emerg. Technol..

[24] González-Aguilar G.A., Wang C.Y., Buta J.G., Krizek D.T. (2008). Use of UV-C irradiation to prevent decay and maintain postharvest quality of ripe “Tommy Atkins” mangoes. Int. J. Food Sci. Technol..

[25] Ayala-Gil M.E., Lozoya-Gloria E. Methods for Increasing the Nutraceutical Content of Perishable Fruits. U.S. Patent.

[26] Oviedo-Solís C.I., Sandoval-Salazar C., Lozoya-Gloria E., Maldonado-Aguilera G.A., Aguilar-Zavala H., Beltrán-Campos V., Pérez-Vázquez V., Ramírez-Emiliano J. (2017). Ultraviolet light-C increases antioxidant capacity of the strawberry (Fragaria × ananassa) in vitro and in high-fat diet-induced obese rats. Food Sci. Nutr..

[27] Institute of Laboratory Animal Resources (1996). Guide for the Care and Use of Laboratory Animals.

[28] Ochoa M.L.I. (2001). Norma Oficial Mexicana (NOM-062-ZOO-1999). Especificaciones Técnicas Para la Producción, Cuidado y uso de los Animales de Laboratorio.

[29] Giampieri F., Forbes-Hernandez T.Y., Gasparrini M., Alvarez-Suarez J.M., Afrin S., Bompadre S., Quiles J.L., Mezzetti B., Battino M. (2015). Strawberry as a health promoter: An evidence based review. Food Funct..

[30] Giampieri F., Alvarez-Suarez J.M., Battino M. (2014). Strawberry and human health: Effects beyond antioxidant activity. J. Agric. Food Chem..

[31] Oviedo-Solís C.I., Cornejo-Manzo S., Murillo-Ortiz B.O., Guzmán-Barrón M.M., Ramírez-Emiliano J. (2018). Strawberry polyphenols decrease oxidative stress in chronic diseases. Gaceta Medica de Mexico.

[32] Giampieri F., Alvarez-Suarez J., Mazzoni L., Forbes-Hernandez T., Gasparrini M., Gonzàlez-Paramàs A., Santos-Buelga C., Quiles J., Bompadre S., Mezzetti B. (2004). Polyphenol-rich strawberry extract protects human dermal fibroblasts against hydrogen peroxide oxidative damage and improves mitochondrial functionality. Molecules.

[33] Forbes-Hernández T.Y., Afrin S., Cianciosi D., Manna P.P., Zhang J., Gasparrini M., Reboredo-Rodriguez P. (2018). Strawberry extract attenuates oxidative stress in 3T3-L1 cells. J. Berry Res..

[34] Mateos R., Lecumberri E., Ramos S., Goya L., Bravo L. (2005). Determination of malondialdehyde (MDA) by high-performance liquid chromatography in serum and liver as a biomarker for oxidative stress. Application to a rat model for hypercholesterolemia and evaluation of the effect of diets rich in phenolic antioxidant. J. Chromatogr. B Anal. Technol. Biomed. Life Sci..

[35] Forbes-Hernández T., Giampieri F., Gasparrini M., Afrin S., Mazzoni L., Cordero M., Mezzetti B., Quiles J., Battino M. (2017). Lipid Accumulation in HepG2 Cells Is Attenuated by Strawberry Extract through AMPK Activation. Nutrients.

[36] Alvarez-Suarez J.M., Dekanski D., Ristić S. (2011). Strawberry polyphenols attenuate ethanol-induced gastric lesions in rats by activation of antioxidant enzymes and attenuation of MDA increase. PLoS ONE.

[37] Morrison C.D., Pistell P.J., Ingram D.K., Johnson W.D., Liu Y., Fernandez-Kim S.O., White C.L., Purpera M.N., Uranga R.M., Bruce-Keller A.J. (2010). High fat diet increases hippocampal oxidative stress and cognitive impairment in aged mice: Implications for decreased Nrf2 signaling. J. Neurochem..

[38] Cobb C.A., Cole M.P. (2015). Oxidative and nitrative stress in neurodegeneration. Neurobiol. Dis..

[39] Valladolid-Acebes I., Merino B., Principato A., Fole A., Barbas C., Lorenzo M.P., García A., Del Olmo N., Ruiz-Gayo M., Cano V. (2012). High-fat diets induce changes in hippocampal glutamate metabolism and neurotransmission. Am. J. Physiol. Endocrinol. Metab..

[40] Sickmann H.M., Waagepetersen H.S., Schousboe A., Benie A.J., Bouman S.D. (2010). Obesity and type 2 diabetes in rats are associated with altered brain glycogen and amino-acid homeostasis. J. Cereb. Blood Flow Metab..

[41] Hao S., Dey A., Yu X., Stranahan A.M. (2016). Dietary obesity reversibly induces synaptic stripping by microglia and impairs hippocampal plasticity. Brain Behav. Immun..

[42] Baldo B.A., Spencer R.C., Sadeghian K., Mena J.D. (2016). GABA-mediated inactivation of medial prefrontal and agranular insular cortex in the rat: Contrasting effects on hunger- and palatability-driven feeding. Neuropsychopharmacology.

[43] Treviño S., Aguilar-Alonso P., Flores Hernandez J.A., Brambila E., Guevara J., Flores G., Lopez-Lopez G., Muñoz-Arenas G., Morales-Medina J.C., Toxqui V. (2015). A high calorie diet causes memory loss, metabolic syndrome and oxidative stress into hippocampus and temporal cortex of rats. Synapse.

[44] Koopman K.E., Booij J., Fliers E., Serlie M.J., la Fleur S.E. (2013). Diet-induced changes in the lean brain: Hypercaloric high-fat-high-sugar snacking decreases serotonin transporters in the human hypothalamic region. Mol. Metab..

[45] Abram V., Berlec B., Ota A., Šentjurc M., Blatnik P., Ulrih N.P. (2013). Effect of flavonoid structure on the fluidity of model lipid membranes. Food Chem..

[46] Goyarzu P., Malin D.H., Lau F.C., Taglialatela G., Moon W.D., Jennings R., Moy E., Moy D., Lippold S., Shukitt-Hale B. (2004). Blueberry supplemented diet: Effects on object recognition memory and nuclear factor-kappa B levels in aged rats. Nutr. Neurosci..

